# Plasma-derived extracellular vesicle proteins as a source of biomarkers for lung adenocarcinoma

**DOI:** 10.18632/oncotarget.20748

**Published:** 2017-09-08

**Authors:** Jody Vykoukal, Nan Sun, Clemente Aguilar-Bonavides, Hiroyuki Katayama, Ichidai Tanaka, Johannes F. Fahrmann, Michela Capello, Junya Fujimoto, Mitzi Aguilar, Ignacio I. Wistuba, Ayumu Taguchi, Edwin J. Ostrin, Samir M. Hanash

**Affiliations:** ^1^ Department of Clinical Cancer Prevention, The University of Texas MD Anderson Cancer Center, Houston, Texas, 77030, USA; ^2^ Department of Biostatistics, The University of Texas MD Anderson Cancer Center, Houston, Texas, 77030, USA; ^3^ Department of Translational Molecular Pathology, The University of Texas MD Anderson Cancer Center, Houston, Texas, 77030, USA; ^4^ Department of General Internal Medicine, The University of Texas MD Anderson Cancer Center, Houston, Texas, 77030, USA; ^5^ McCombs Institute for the Early Detection and Treatment of Cancer, The University of Texas MD Anderson Cancer Center, Houston, Texas, 77030, USA

**Keywords:** extracellular vesicles and exosomes, proteomics, liquid biopsy, lung cancer, biomarker discovery

## Abstract

Exosomes and other extracellular vesicles (EVs) have been implicated as mediators of intercellular communication. Their release into the circulation has the potential to inform about tumor status. In-depth proteomic characterization of plasma-derived EVs has been limited by challenges in isolating EVs from protein-abundant biological fluids. We implemented a novel single-step density gradient flotation workflow for efficient and rapid isolation of highly enriched circulating EVs from plasma. Mass-spectrometry analysis of plasma EVs from subjects with lung adenocarcinoma and matched controls resulted in the identification of 640 proteins. A total of 108 proteins exhibited significant (*p*<0.05) differential expression in vesicle preparations derived from lung adenocarcinoma case plasmas compared to controls, of which 43 were also identified in EVs from lung adenocarcinoma cell lines. Four top performing EV-associated proteins that distinguished adenocarcinoma cases from controls, SRGN, TPM3, THBS1 and HUWE1, yielded a combined area under the receiver operating characteristic curve (AUC) of 0.90 (95% CI = 0.76-1). Our findings support the potential of EV derived proteins as a source of biomarkers that complement other approaches for tumor assessment.

## INTRODUCTION

Extracellular vesicles are cell-derived structures ranging in diameter from tens of nanometers to a few micrometers that have been described in cell culture supernatants as well as plasma, serum and other body fluids [1]. EVs derived from tumors and released into the circulation have the potential to yield biomarkers for minimally-invasive liquid biopsy applications, including cancer detection, tumor molecular profiling and disease monitoring [2, 3]. EVs contain phospholipids and membrane micro-domains, such as lipid rafts and caveolae, and molecular cargo including proteins, nucleic acids and metabolites [4]. Vesicle-mediated molecular exchanges have been implicated as contributing to hallmarks of cancer including deregulation of cellular energetics and metabolism, maintenance of proliferative signaling, apoptosis resistance, activation of metastasis and invasion, immunomodulation and angiogenesis [5–10].

While EVs have thus far predominantly been considered in the context of exosomes, it is now evident that cells produce and exchange an array of phospholipid-protein complexes of different sizes and composition (e.g., microvesicles, microparticles, exosomes, ectosomes, oncosomes, and lipoproteins) that play various roles in packaging and trafficking molecular cargoes between cells and their milieu [3, 11]. Strictly defined, exosomes are extracellular vesicles 30–150 nm in diameter that originate from multi-vesicular endosomes (MVEs) [3, 11, 12]. The various circulating vesicle populations often co-isolate due to intersecting physical properties, and the term *exosome* has been employed more generally in some studies, being used to denote small EVs without definitive discrimination according to origin [3]. Markers for the analysis and isolation of different EV populations are yet to be fully elucidated and defined, especially for bio-fluid-derived EVs [11].

Comprehensive exploration of the proteome of bio-fluid-derived EVs has been limited due to the difficulty in isolating circulating vesicles from biological fluid specimens, notably plasma, with sufficient yield and purity to allow for in-depth protein profiling. A principal challenge is in discriminating *bona fide* vesicle-associated cargo proteins from non-vesicular proteins that co-fractionate with preparations of exosomes and extracellular vesicles [13]. To address this challenge we developed a single-step density gradient flotation approach to facilitate efficient isolation of circulating EVs from background soluble proteins and protein aggregates. In the present study we compared this approach to ultracentrifugation pellet/wash techniques using transmission electron microscopy, nanoparticle tracking analysis and immunoblotting and demonstrated improved yields of exosome-sized particles, marked depletion of non-vesicle-associated proteins and favorable particle purity metrics.

Using a nested case-control study, we performed in-depth proteomic profiling of plasma-derived EVs from subjects with early stage lung adenocarcinoma and matched controls to identify differentially expressed proteins. We compared these with profiles of unfractionated plasmas as well as EVs derived from conditioned media of lung adenocarcinoma cell lines. Specific characterization of plasma-derived EVs revealed EV-associated proteins not previously found in comprehensive proteomic analyses of lung cancer, lung disease and control plasma specimens. In addition, many previously identified plasma proteins were found to be markedly enriched in EVs compared to unfractionated plasma. Our findings indicate that plasma EVs can harbor biomarker information that may be missed by conventional profiling of total plasma and support the utility of mining the plasma EV proteome as a source of lung cancer biomarkers.

## RESULTS

### Isolation of extracellular vesicles from plasma

**Figure 1:**
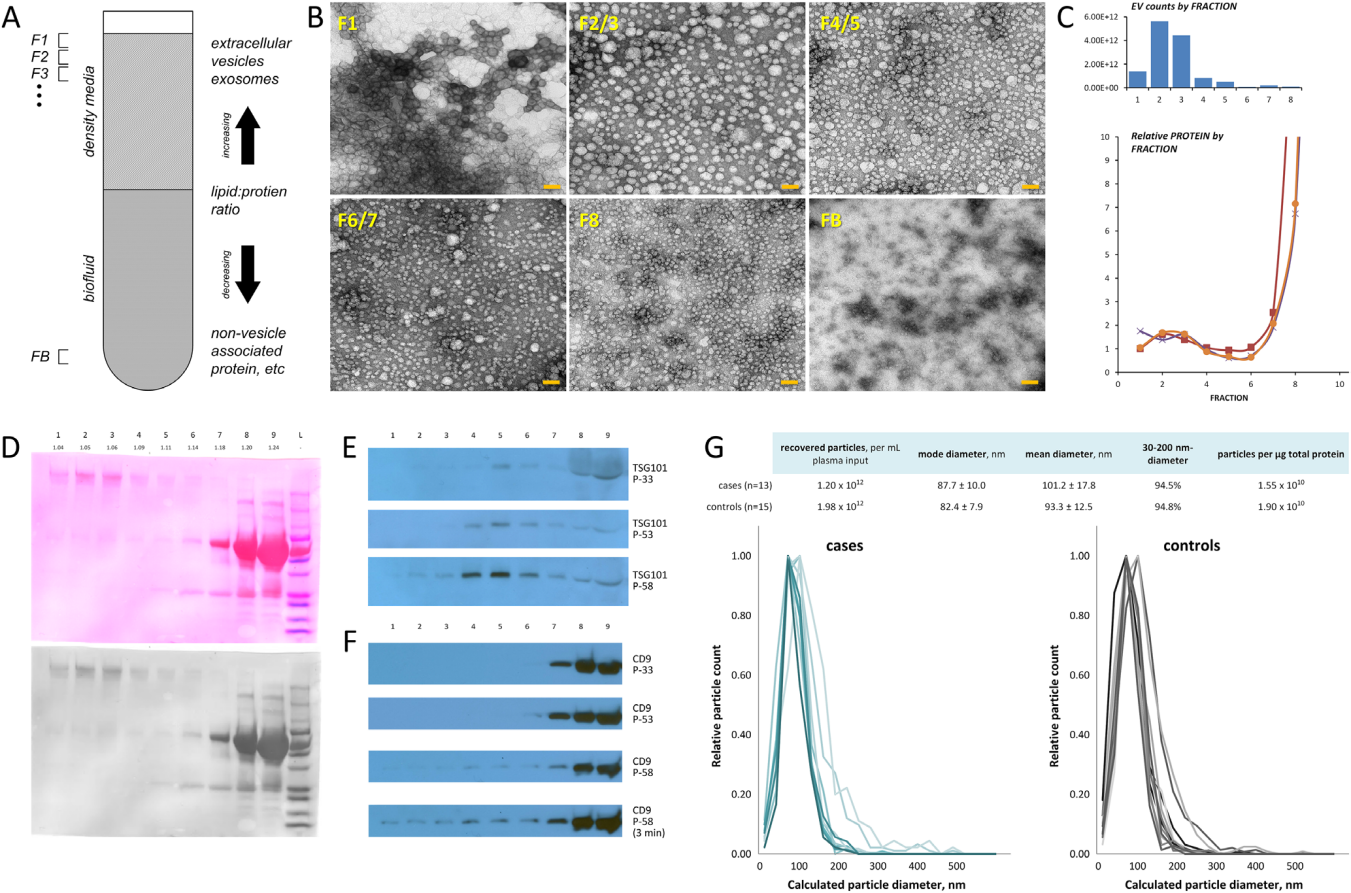
Isolation of extracellular vesicles from plasma **(A)** Gradient flotation principle. Plasma samples are density-adjusted and overlaid with lower-density fractionation media to form single- or multi-step gradients; circulating extracellular vesicles are separated according to density by ultracentrifugation. Non-vesicle-associated proteins exhibit negative buoyancy and sediment downward; vesicle complexes float upward to neutral (or minimal) density and are collected from the upper zones of the overlay. **(B)** Transmission electron microscopy images of plasma-derived EVs obtained from multi-step density fractionation of plasma (scale bar, 100 nm). Images are arrayed in order of increasing fraction density; vesicle-depleted plasma is shown in *lower-right* image (FB). Fraction numbers correspond with those specified in Figure 1. **(C)** Relative distribution of exosome-sized particles (*upper graph*) and protein abundance (*lower graph*) in vesicle populations obtained from multi-step density fractionation of plasma. Fractions of equal volume were collected from the top of the tube proceeding downward for quantification by nanoparticle-tracking and protein assay. Fraction numbers correspond to relative position specified in A and shown in B. **(D)** Ponceau S staining for total protein content of density fractionated vesicle harvests, indicating relative protein abundance in upper fractions (F1-F8) and vesicle-depleted plasma (FB). Immunoblot staining of fractionated EVs from three different plasma samples for **(E)** endosomal sorting marker TSG101 and **(F)** cell surface glycoprotein marker CD9, showing relative expression of these markers according to EV buoyant density as well as in vesicle-depleted plasma (lane 9). **(G)** Nanoparticle-tracking analysis of EV preparations from adenocarcinoma case and matched control plasmas, indicating size distribution of isolated particles. Size and yield data are summarized (*upper*). Cohort samples were prepared by ultracentrifugation flotation through a single-step density-overlay (*ρ* = 1.14 g/mL).

EVs were isolated from plasma by ultracentrifugation flotation though a multi-step density-gradient overlay. Fractions of equal volume were collected from the top of the tube proceeding downward (Figure 1A), yielding vesicle subpopulations with increasing protein:lipid ratio and buoyant densities spanning the range ≤ 1.04-1.32 g/mL. Transmission electron microscopy (TEM) analyses confirmed enrichment of typical exosome-sized (30-200 nm-diameter) vesicles in harvest fractions (Figure 1B, upper and left-most images and Supplementary Figure 2: F1-F8) as well as their absence in the depleted plasma inputs following EV isolation (Figure 1B, lower-right: FB). Nanoparticle-tracking analyses (Particle Metrix, GmbH) were used to quantify the number and size distribution of vesicles in each fraction (Figure 1C, upper), revealing the highest enrichment of exosome-sized particles in fractions 2 and 3 and minimal representation of exosomes in fractions having density ≥ 1.18 (F7-F8).

Protein abundance (Figure 1C, lower) and expression of canonical exosome markers in the fractionated vesicle subpopulations of plasma-derived EVs were also evaluated. Ponceau S staining (Figure 1D) of transfer membranes prepared from equal volume aliquots of each fraction revealed the highest protein abundance in fractions containing particles with density > 1.20 g/mL (F8/9), and relatively low total protein in the lower density fractions (F4-6), with evidence of enrichment of high molecular weight proteins in the least dense fractions (F1-3) containing particles of density < 1.09 g/mL. Immunoblot staining for endosomal sorting marker TSG101 (Figure 1E) revealed enrichment in vesicles of intermediate density 1.09-1.14 g/mL (F4-6) and additional expression in vesicle-depleted plasma fractions. Of note, although cell surface glycoprotein marker CD9 was detected in vesicle-enriched, low and intermediate fractions 1 through 5 (Figure 1F) it was more abundant in less buoyant (≥ 1.20 g/mL), protein-rich fractions and vesicle-depleted plasma. These data indicate diversity of plasma-derived circulating lipid-protein particles within the size range and density of exosomes whose marker expression is somewhat distinct from that typically reported for EVs obtained from cell conditioned media.

In order to carry out comprehensive proteomic exploration of plasma-derived EVs exhibiting various exosomal features within the context of an efficient workflow, we employed ultracentrifugation flotation through a single-step density-overlay (*ρ* = 1.14 g/mL) for isolation of EVs from a cohort of lung adenocarcinoma patient (*n* = 13) and control (*n* =15) plasmas (Table 1). This enabled concurrent recovery of EV subpopulations F1-F6, comprising ~90% of the exosome-sized particles in plasma and including those bearing high expression of canonical marker TSG101, while minimizing sample input requirements and processing time. Nanoparticle-tracking analyses verified enrichment of exosome-sized particles in the plasma-EV isolates (Figure 1G). The average modal size of the particles isolated from adenocarcinoma case plasma was 88 ± 10 nm, 94.5% of which were exosome-sized and the average recovery was 1.20 × 10^12^ particles per ml of plasma input. In the control plasma, the average modal size of the isolated particles was 82 ± 8 nm, 94.8% were exosome-sized and the average recovery was 1.98 × 10^12^ particles per ml of plasma input. The purity of the vesicle preparations was evaluated according to the standardized reporting criterion proposed by Webber and Clayton, wherein relative sample purity is defined as the ratio of total nanoparticle counts to total sample protein, expressed in particles per μg. The average purity of the respective patient and control samples was 1.6 × 10^10^ and 1.9 × 10^10^ particles per μg of total protein recovered, indicating an ~3-fold improvement over typical pellet/wash approaches for the isolation of EVs from bio-fluids [13] (Supplementary Figure 3).

**Table 1:** Clinicopathological features of lung adenocarcinoma patient and matched control discovery cohort

A. Summary									
	Total			Cases			Controls		
**N**	28			13			15		
**Sex, n (%)**									
**Male**	10	(35.7)		5	(38.5)		5	(33.3)	
**Female**	18	(64.3)		8	(61.5)		10	(66.7)	
**Age, y**	63.7	±	7.6	63.5	±	8.3	63.9	±	7.1
**Smoking, n (%)**									
**Never**	3	(10.7)		3	(23.1)		0	(0.0)	
**Former**	13	(46.4)		8	(61.5)		5	(33.3)	
**Current**	12	(42.9)		2	(15.4)		10	(66.7)	
**Stage, n (%)**									
**I**				6	(46.2)				
**II**				7	(53.8)				

B. Lung adenocarcinoma cases									
S	Age	Sex	Smoking status	Pack years	Tumor size	T	N	M	Stage
**1**	65	M	former	2.2	4	T2a	N1	M0	IIA
**2**	58	F	former	< 2	3.5	T2a	N0	M0	IB
**3**	63	M	former	58	2.2	T2a	N0	M0	IB
**4**	55	F	former	4	2.6	T1b	N1	M0	IIA
**5**	63	M	current	76	2.0 & 0.7	T3	N0	M0	IIB
**6**	60	F	never	n/a	2.5	T1b	N0	M0	IB
**7**	76	F	former	9	2.1	T1b	N0	M0	IA
**8**	49	F	current	50	1.8	T1a	N0	M0	IA
**9**	60	M	former	8	3.3	T2a	N1	M0	IIA
**10**	63	F	former	40	3.3	T2a	N1	M0	IIA
**11**	63	F	never	n/a	3.2	T2a	N1	M0	IIA
**12**	68	M	former	7	1.2	T2a	N0	M0	IB
**13**	81	F	never	n/a	8.0 & 1.0	T3	N0	M0	IIB

C. Controls									
S	Age	Sex	Smoking status	Pack years
**1**	62	F	current	40
**2**	58	F	former	44
**3**	68	M	current	55
**4**	60	M	former	33
**5**	76	F	current	118
**6**	73	F	current	25
**7**	72	F	current	26
**8**	51	F	current	35
**9**	53	F	current	37
**10**	63	F	current	71
**11**	70	F	former	40
**12**	60	F	former	45
**13**	63	M	current	30
**14**	63	M	current	46
**15**	66	M	former	22

### Profiling of plasma-derived extracellular vesicle proteins

**Figure 2:**
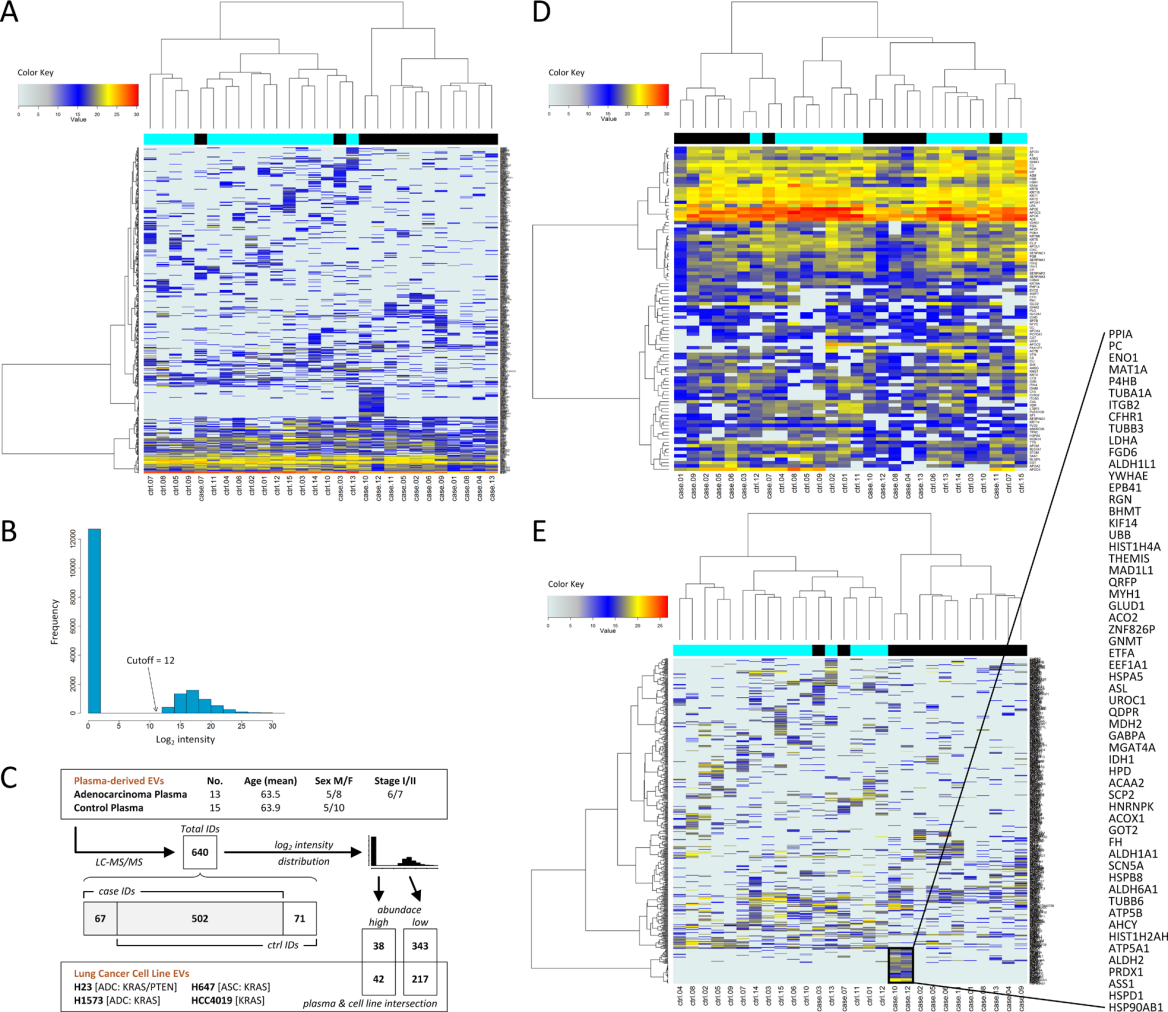
Hierarchical clustering analyses of plasma-derived EV proteins Pearson's correlation coefficient and Ward's method were used. Lung adenocarcinoma cases are in black; disease free controls are in cyan. **(A)** Intensity heat map of all proteins identified by LC MS/MS proteomics. **(B)** Bimodal distribution of log_2_ relative intensities for identified proteins reveals high and low abundance protein groups. **(C)** Integration and filtering scheme for plasma- and cell line-derived EV datasets. **(D)** Intensity heat map of high abundance protein group. **(E)** Intensity heat map of low abundance protein group; a cancer-related cluster is highlighted.

LC MS/MS proteomic profiling of lung adenocarcinoma patient and control plasma-derived EVs yielded identification of 640 vesicle-associated proteins of which 67 were unique to cases, 502 were identified in both cases and controls and 71 were unique to controls. We performed unsupervised hierarchical clustering to identify potential EV-associated tumor signatures (Supplementary Figures 4-6); Figure 2A depicts an intensity heatmap of all identified proteins. Due to data sparsity and bimodal distribution, we considered the data in two specific subgroups according to a log_2_ intensity cutoff value of 12 (Figure 2B). Based on the average intensity for each identified protein, we classified 80 proteins that were above the cutoff value as *high abundance*, and 560 proteins equal to or below the cutoff as *low abundance*. High abundance proteins included typical plasma proteins such as apolipoproteins, complement components and keratins (Figure 2D). Gene ontology (GO) enrichment analysis [14] of the high abundance proteins (Table 2C) revealed that the top three cellular component terms for the group were: *blood microparticle*, *extracellular space* and *extracellular exosome* (*FDR* = 4.0E-41 – 1.4E-78). The top three cellular component terms enriched in the low abundance proteins (Table 2A) were: *extracellular exosome*, *extracellular region*, and *membrane-bounded vesicle* (*FDR* = 1.7E-30 – 3.2E-34).

**Table 2:** GO enrichment analysis, cellular component and Ingenuity Pathway Analysis, diseases and functions for low and high abundance proteins conveyed by plasma-derived extracellular vesicles from lung adenocarcinoma cases and disease-free controls

A. GO enrichment analysis, cellular component: low abundance (560)			
Pathway ID	Pathway description	Observed gene count	False discovery rate
GO.0070062	extracellular exosome	185	3.16E-34
GO.0044421	extracellular region part	210	7.91E-31
GO.0031988	membrane-bounded vesicle	201	1.69E-30
GO.0031982	vesicle	202	3.16E-29
GO.0005576	extracellular region	221	1.97E-25

B. Pathway analysis, diseases and functions: low abundance case:control ratio >1.3-fold (203)		
Diseases and Bio Functions, Categories	*p*-Value range	# Molecules
**Diseases**		
Cancer	2.9E-03 – 1.3E-11	192
Organismal Injury and Abnormalities	2.9E-03 – 1.3E-11	194
Respiratory Disease	2.9E-03 – 1.3E-11	66
**Bio Functions**		
Cell Death and Survival	2.7E-03 – 2.1E-10	90
Cellular Movement	2.9E-03 – 1.4E-09	52
Carbohydrate Metabolism	1.8E-03 – 2.2E-07	39

C. GO enrichment analysis, cellular component: high abundance (80)			
Pathway ID	Pathway description	Observed gene count	False discovery rate
GO.0072562	blood microparticle	43	1.38E-78
GO.0005615	extracellular space	57	1.40E-51
GO.0070062	extracellular exosome	63	4.04E-41
GO.0044421	extracellular region part	64	1.97E-35
GO.0005576	extracellular region	64	6.03E-31

D. Pathway analysis, diseases and functions: high abundance (80)		
Diseases and Bio Functions, Categories	*p*-Value range	# Molecules
**Diseases**		
Metabolic Disease	1.1E-04 – 3.7E-23	43
Neurological Disease	1.4E-04 – 1.1E-20	43
Gastrointestinal Disease	1.1E-04 – 6.1E-16	71
**Bio Functions**		
Lipid Metabolism	1.3E-04 – 5.4E-15	38
Molecular Transport	1.4E-04 – 5.4E-15	47

### Patient-derived and control EVs manifest different protein cargoes

Unsupervised hierarchical clustering was conducted to evaluate the distribution of low abundance group proteins between cancer cases and controls. Two main clusters were observed that largely represented differences between subjects with adenocarcinoma relative to controls (Figure 2E), indicating that adenocarcinoma-associated EVs exhibited differential signatures compared to control EVs. Ingenuity pathway analysis (IPA) of the 203 low abundance group proteins with > 1.2-fold higher differential expression in adenocarcinoma patient plasma-derived EVs relative to controls revealed two of the top three disorders were cancer (192 proteins) and respiratory disease related (66 proteins) (*p*-value range: 2.9E-03 – 1.3E-11). The top three molecular and cellular functions were related to cell death and survival (90 proteins), cellular movement (52 proteins), and carbohydrate metabolism (39 proteins) (*p*-value range: 1.8E-03 – 2.1E-10). The top two scoring IPA networks showed good integration of these disease and functional nodes (*P-score*=10^-39^ and *P-score*=10^-37^), based on relationships annotated as experimentally observed in the Ingenuity Knowledge Base (Table 2B; Supplementary Figure 1). In contrast, the high abundance EV proteins were associated by IPA with lipid metabolism and molecular transport functions (Table 2D).

**Figure 3:**
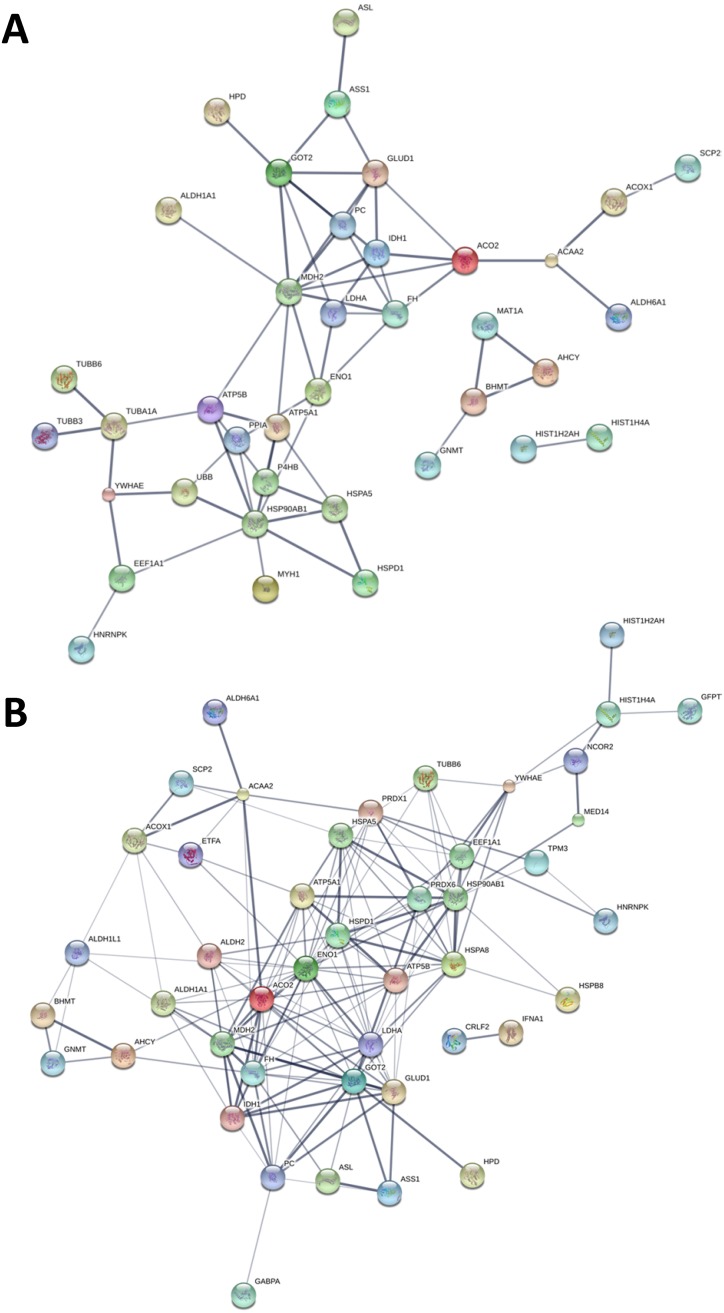
STRING Protein-protein interaction analyses **(A)** STRING PPI network connectivity of lung adenocarcinoma plasma-derived EV protein cluster highlighted in Figure 2E. Network contains 62 edges (vs. 11 expected edges); enrichment p-value< 0.001. **(B)** STRING PPI network connectivity for adenocarcinoma case EV-associated proteins exhibiting > 2-fold higher mean expression compared to controls. Network contains 75 nodes with 160 edges (vs. 38 expected edges); clustering coefficient: 0.742; enrichment p-value < 0.001. Confidence score threshold was set at 0.7 (high) for both analyses.

Intensity heat map analysis of the low abundance proteins revealed a prominent cluster consisting of 58 proteins within a subset of subjects in the cancer group (Figure 2E). We subjected this cluster to protein-protein interaction (PPI) analysis using the Search Tool for the Retrieval of Interacting Genes (STRING) database [14] (Supplementary Figures 7 and 8). The resulting network (Figure 3A) comprised significantly more interactions than expected for a random set of similar size drawn from the genome, implying functional intersection of the EV proteins represented in the cancer group cluster (expected number of edges: 11; actual number of edges: 62; enrichment *p*-value < 0.001; confidence score: high, 0.7).

### Cancer context and network features of plasma-derived EV-associated proteins

We compared the proteins that were differentially expressed in adenocarcinoma patient-derived EVs, with EVs isolated from the conditioned media of four lung adenocarcinoma cell lines (H23, H647, H1573, HCC4019). Of the total 569 proteins identified in patient plasma EVs, 238 (42%) were also identified in the cell line EVs; for the low abundance proteins identified in the patient plasma EVs, 196 of 489 (40%) were concordant with those identified in the cell line EVs. Of the 196 case / cell line intersecting proteins, 74 (38%) had >2.0-fold higher mean expression in the cases with 34 being uniquely identified in the case, but not control EVs (Supplementary Table 1).

We integrated the *in vivo* and *in vitro* plasma- and cell line-derived EV protein datasets (Figure 2C) and explored network connectivity and ontological intersection of overlapping features to ascertain biological aspects of the circulating vesicle proteome in the context of lung adenocarcinoma. Using the STRING protein-protein interaction database [14] we analyzed the network connectivity between adenocarcinoma case EV-associated proteins exhibiting > 2-fold higher mean expression in the cases. Using an interaction score threshold of 0.7 (high confidence), the STRING PPI analysis (Figure 3B) yielded a highly clustered network (clustering coefficient: 0.742) containing 75 nodes with 160 edges (expected number of edges: 38), indicating significantly more interaction than expected for a random set of similar size drawn from the genome (enrichment *p*-value < 0.001). Enrichment analysis (Supplementary Table 2) reveals significant GO cellular component terms in the network include *extracellular vesicle* and *membrane bounded vesicle*, as well as *mitochondrion* and *mitochondrial matrix* components (FDR = 8.6E-06 – 1.4E-12); GO molecular function terms for the network include *catalytic* and other *enzymatic activities* and *small molecule*, *nucleic acid*, *protein* and *ubiquitin protein ligase binding* (FDR = 2.4E-02 – 6.7E-04). KEGG (Kyoto Encyclopedia of Genes and Genomes) [15] pathways with significance in the network include metabolic, biosynthetic, and antigen processing and presentation pathways (FDR = 4.1E-02 – 5.4E-11).

### Potential of plasma-derived EV-associated proteins as a source of lung adenocarcinoma biomarkers

The value of exploring plasma EV-associated proteins is in providing access to markers not otherwise readily detected in plasma. Using previously acquired data from comprehensive proteomic profiling of multiple (26) paired lung cancer, lung disease and control plasma specimens, we compared the identified plasma EV-associated proteins against those found in unfractionated plasma. Of the 569 proteins identified in the patient plasma EVs, 231 (41%) had not been previously identified in unfractionated plasma based on our historical data, indicating that plasma EVs can harbor additional information that may be missed by conventional profiling of total plasma.

**Figure 4:**
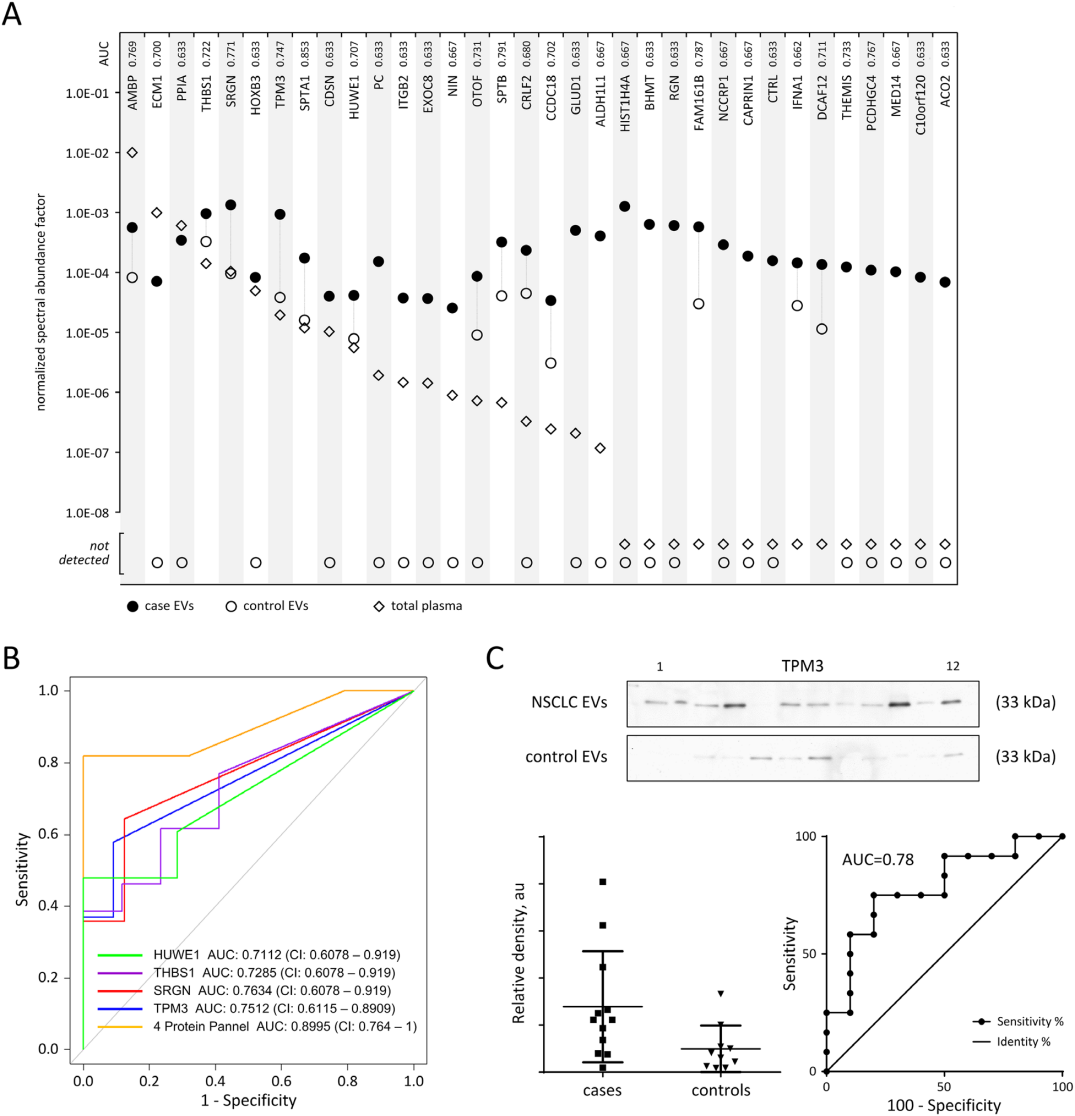
Enrichment of tumor proteins in plasma-derived EVs and classifier performance **(A)** Differential enrichment of EV proteins was evaluated for proteins with ≥ 3-fold case:control expression and *p* < 0.05. The average normalized spectral abundance factor (NSAF) for each protein was calculated from respective LC-MS/MS proteomic analyses of adenocarcinoma case plasma-derived EVs (●) black circle; disease free control plasma-derived EVs (○) open circle; and unfractionated plasma from paired cancer, lung disease and control specimens (◊) diamond. **(B)** Receiver operating characteristic curve analyses of top performing plasma EV protein markers SRGN (red), TPM3 (blue), THBS1 (violet), and HUWE1 (green). The four marker combination (yellow) yielded an AUC of 0.8995. **(C)** Immunoblot analysis of plasma-derived extracellular vesicles from adenocarcinoma cases and matched controls to demonstrate marker performance in immunoassay. Marker abundance as quantified by densitometry of western blot, lower left; ROC curve analysis of TPM3 performance based on western blot data, lower right.

Within our dataset, 34 vesicle-associated proteomic features exhibited statistical significance of *p* < 0.05 and 3-fold or higher (21 were case-exclusive) increased expression in the adenocarcinoma case compared to control plasma-derived EVs. We determined whether these 34 proteins were enriched in EVs relative to unfractionated plasma by contrasting the average normalized spectral abundance factor (NSAF) [16] for each protein within the respective proteomic analyses (Figure 4A). Of the 34 proteins, 26 exhibited > 10-fold higher average spectral abundance in the case vesicles compared to unfractionated plasma. Notably, 14 of these vesicle-associated proteins were among those not previously identified in our comprehensive proteomic profiling of paired cancer, lung disease and control plasma specimens. Of the 34 proteins of interest, 13 exhibited concordant expression in both the plasma and lung cancer cell-line-derived EVs (Table 3). To characterize the potential utility of circulating EV associated proteins as biomarkers for lung adenocarcinoma, we performed receiver operating characteristic (ROC) curve analyses to determine classifier performance. AUCs of the 34 proteins demonstrated performance values ranging from 0.63-0.77 (Figure 4A). Top performing protein markers consisted of secretory vesicle proteoglycan Serglycin (SRGN); actin filament-binding protein Tropomyosin alpha-3 chain (TPM3); adhesive glycoprotein Thrombospondin-1 (THBS1) and E3 ubiquitin-protein ligase HECT, UBA and WWE domain-containing protein 1 (HUWE1) (Figure 4B), all of which were also detected in lung cancer cell line derived EVs. The combination of these four markers yielded an AUC of 0.8995 (confidence interval = 0.764-1) (Figure 4B). As a test of our findings, we assessed TPM3 performance using immunoblots of EVs from cases and controls (Supplementary Table 3). Consistent with our proteomics findings, TPM3 was elevated in EVs from cases relative to controls and yielded a classification performance AUC of 0.78 (Figure 4C).

**Table 3:** Proteins of translational interest enriched in lung adenocarcinoma EVs

Gene	Avg(ctrl)	Avg(case)	Fold change(case:ctrl)	AUC	*p*-value
SRGN	8.69E+03	4.61E+04	5.30	0.771	0.005
TPM3	6.05E+02	2.67E+04	44.10	0.747	0.005
THBS1	5.37E+03	2.83E+04	5.27	0.722	0.026
HUWE1	1.12E+04	9.36E+04	8.36	0.707	0.027
CCDC18	6.38E+03	6.24E+04	9.77	0.702	0.016
ALDH1L1	0.00E+00	1.14E+05	^***^	0.667	0.018
HIST1H4A	0.00E+00	6.60E+04	^***^	0.667	0.018
NCCRP1	0.00E+00	1.06E+04	^***^	0.667	0.018
MED14	0.00E+00	7.00E+03	^***^	0.667	0.018
BHMT	0.00E+00	7.71E+04	^***^	0.633	0.038
GLUD1	0.00E+00	5.24E+04	^***^	0.633	0.038
PPIA	0.00E+00	1.89E+04	^***^	0.633	0.038
EXOC8	0.00E+00	5.58E+03	^***^	0.633	0.038

Filtering criteria: case:control ratio >2-fold; *p* < 0.05; plasma/cell-line concordance.

## DISCUSSION

We have investigated features of the tumor proteome that are conveyed by circulating vesicles in the context of lung adenocarcinoma. Proteomic exploration of the plasma EV compartment revealed differentially expressed proteins that offer circulating evidence of tumorigenic reprogramming including proteins related to altered metabolism (GLUD1, ALDH1L1, BHMT) [17–19], protein fate and trafficking (HUWE1, EXOC8, NCCRP1) [20–23], cytoskeletal remodeling (TPM3) [24], transcriptional regulation (HIST1H4A, MED14) [25, 26] and tumor stemness (SRGN) [27]. Importantly, our findings indicate that isolation of EVs from plasma allows for enrichment of potential tumor-derived proteomic signatures that exhibit diagnostic value and may otherwise be missed by conventional profiling of complete plasma via mass spectrometry.

Identification of vesicle-associated proteins in adenocarcinoma case plasmas with known oncogenic roles supports the potential of plasma-derived EVs for liquid biopsy based query of tumor status. SRGN is overexpressed in a number of cancers and elevated cellular SRGN has been correlated with poor survival and recurrence in nasopharyngeal and hepatocellular carcinomas. In primary non-small cell lung cancers, SRGN is overexpressed by both carcinoma and stromal cells, and SRGN has been shown to induce lung cancer cell stemness and promote NSCLC cell migration, invasion and metastatic colonization [27]. Altered expression of tropomyosin isoforms is concomitant with pronounced rearrangements in actin cytoskeleton architecture and has been observed as a patent feature in a number of malignancies. TPM3 specifically has been implicated as an oncogene that modulates focal adhesion stability as well as cell migration, invasion and proliferation [24]. THBS1 is a multifunctional glycoprotein found to be overexpressed in the tumor microenvironment, where it regulates tumor cell adhesion and migration, metastasis and angiogenesis [28]. THBS1 is highly expressed in the stroma of angiogenic non-small-cell lung tumors that also exhibit increased expression of genes for membrane vesicles, integrins, remodeling, angiogenesis and apoptosis [29]. The observed relationship between THBS1 and membrane vesicle aparatus in angiogenic tumor types provides mechanistic insight into the finding of EV-associated THBS1. The E3 ubiquitin (Ub) ligase HUWE1 has been implicated in tumor formation, cell migration and invasion and is significantly overexpressed in a number of epithelial tumors, including lung carcinoma [30]. Alterations to the ubiquitylation machinery, a principal regulator of protein degradation, are found in a number of human cancers. In lung carcinoma cells, aberrant HUWE1-mediated ubiquitylation has been shown to contribute to enhanced cell-cell adhesion disassembly, motility, and invasiveness [20].

In exploring the potential tumor cell source of these differentially expressed proteins, we also characterized the proteome of secreted extracellular vesicles derived from the conditioned media of multiple non-small cell lung adenocarcinoma cell lines. Intersection and bioinformatic consideration of these *in vivo* and vitro data robust representation of extracellular exosome and membrane-bounded vesicle cellular components; pathways associated with cancer and organismal injuries and abnormalities; and biological processes related to metabolism and biosynthesis, regulation of protein localization and protein quality control.

A major bottleneck for interrogating extracellular vesicles and exosomes in research or clinical domains has been in obtaining enriched preparations of vesicles that contain oncogenic cargo or signatures of the tumor cells from which the originate. Existing approaches often yield mixtures of extracellular vesicles and non-vesicular protein and nucleic acid “cargoes” that confound resultant profiling data [11, 13]. Sandfeld-Paulsen and colleagues have applied a targeted immunoaffinity capture approach using an extracellular vesicle microarray of 49 antibodies to provide phenotyping of plasma-derived EVs for known exosomal and cancer cell markers [31, 32]. In pursuit of a means for enriching circulating extracelluar vesicles from bio-fluids for untargeted proteomic discovery studies that would also anticipate clinical implementation, we developed a streamlined single-step gradient flotation procedure to eliminate indeterminate non-vesicular protein background and facilitate comprehensive profiling of *in vivo* vesicle-associated proteomes.

We observed in our study that the majority of plasma-derived circulating particles within the size range and density of exosomes are not specifically enriched in canonical exosome markers, but are phospholipid-protein lipoprotein complexes with size, density, morphology and nano-tracking properties akin to those reported for exosomes. This parallels emerging data regarding the proteomic diversity of lipoproteins [33, 34], indicating broad function beyond lipid transport and metabolism and suggests roles for lipoproteins that may intersect with those observed and postulated for extracellular vesicles *in vivo*. These include immune modulation, homeostatic regulation, acute-phase response, and morphogen transport. Further investigations are needed to reconcile these entities with their specific functions.

## MATERIALS AND METHODS

### Isolation of EVs by density gradient flotation

All human blood samples were obtained from MD Anderson biospecimen resources following institutional review board approval and written informed consent from study participants. Whole blood samples were collected by venipuncture from lung adenocarcinoma patients and lung cancer free matched control subjects. Plasma was prepared from EDTA-treated whole blood by two successive 12 min room temperature (RT) centrifugation runs at 1,200 x*g*, without braking. Microvesicles were depleted from plasma samples by centrifugation at 2000 x*g* for 20 min followed by 16,500 x*g* for 30 min; the resulting supernatant was additionally filtered through a pre-wetted 0.22 μm vacuum filter (Steriflip SCGP00525, Millipore, Billerica MA). Microvesicle-depleted plasma was densified by mixing with OptiPrep iodixanol solution (Sigma D1556) to a final density of 1.16-1.30 g/mL. This was loaded into the bottom of a polycarbonate ultracentrifuge tube (Seton Scientific, Petaluma, CA) and overlaid with 0.5-2mL aliquots of iodixanol/PBS solution in the 1.20-1.01 g/mL (35-0% wt/vol) range, proceeding from the highest to lowest density to form a single- or multi-step density fractionation gradient as needed. Ultracentrifugation was performed for at 100,000 ×*g* for 4 hrs at 8°C. Vesicles were collected to from the top of the tube, proceeding downward to recover volume equal to 90% of overlaid gradient volume. Density of harvested fractions was assessed against a standard curve based on sample absorbance at 250 nm using a NanoDrop microvolume spectrophotometer (Thermo Fisher Scientific, Wilmington, DE). Vesicle harvests were stored at -80°C.

### Transmission electron microscopy (TEM)

Extracellular vesicle aliquots were fixed in 2% paraformaldehyde; 5 μL of EV suspension was then applied to each formvar/carbon-coated 200 mesh nickel grid and allowed to adsorb for 20 min. The grids were washed twice on 100 μL drops of PBS, followed by eight washes with deionized water. Uranyl acetate (2%) was used as a counterstain; after 1 min of staining, excess uranyl acetate was blotted from the grid edge with Whatman No. 1 filter paper, and the grids were air-dried. EM grids and reagents were from EMS (Hatfield, PA). Imaging was performed using a JEM1010 TEM (JEOL, Peabody, MA) at an accelerating voltage of 80 kV. Digital images were taken with AMT imaging software (Advanced Microscopy Techniques Corp, Danvers, MA).

### Particle size distribution and quantification

Samples of extracellular vesicles were quantified via Brownian diffusion size analyses using ZetaView Nanoparticle-tracking analysis (NTA) instrumentation (Particle Metrix, Meerbusch, Germany). Sample aliquots were diluted 10^2^-10^6^-fold to achieve optimal concentration for analysis; 1.0 mL of diluted sample was used for each analysis. Light scattering of individual particles in solution was digitally recorded, particle trajectory and displacement were automatically analyzed by image analysis tracking software, and the particle-size distribution was determined from the observed Brownian motion of individual particles according to the Stokes-Einstein relationship.

### Protein quantitation, SDS-PAGE and western blot assay

Protein quantification was performed using Pierce BCA Protein Assay (Thermo Fisher Scientific, Rockford, IL) according to the manufacturer's recommended Microplate assay procedure. Absorbance was measured with a SpectraMax M5 multi-mode microplate reader using SoftMax Pro data acquisition and analysis software (MolecularDevices, Sunnyvale CA). Vesicle isolates were denatured in 4x Laemmli sample buffer at 100°C for 10 minutes. Proteins were separated using 4-15% sodium dodecyl sulfate polyacrylamide gel electrophoresis in Tris/Glycine/SDS running buffer and transferred to Immun-Blot PVDF membrane (all reagents and supplies from Bio-Rad, Hercules, CA). Immunoblotting was performed with the following primary antibodies: CD9 (EXOAB-CD9A-1, System Biosciences, Palo Alto, CA); TSG101 (ab125011, Abcam, Cambridge, MA); and TPM3 (H00007170-M02, Novus Biologicals, Littleton, CO). Blots were washed and incubated with appropriate HRP-conjugated secondary antibodies (Amersham ECL, GE, Uppsala, Sweden) and detected using Pierce ECL western blotting substrate (Thermo Fisher Scientific) with chemiluminescence-optimized autoradiography film. Densitometry analysis was performed on digitized images using NIH ImageJ image processing and analysis software.

### Mass spectrometry analysis of plasma-derived EVs

Vesicle harvests were exchanged into Dulbecco's PBS using Zeba spin desalting columns, 7K MWCO (Thermo Fisher Scientific) and denatured in 4x Laemmli sample buffer with addition of SDS at 5% final concentration. Proteins were separated using 4-15% sodium dodecyl sulfate polyacrylamide gel electrophoresis to decomplex the sample; after staining with Coomassie Brilliant Blue, respective gel lanes were divided into 10 fractions each for individual profiling. Gel fractions were minced into 1 mm^3^ cubes; destained for 2 hrs in a 1:1 mix of 100 mM NH_4_HCO_3_/acetonitrile; alkylated for 30 min with 0.154% DTT in 100 mM NH_4_HCO_3_; additionally alkylated for 30 min with 0.271% acrylamide in 100 mM NH_4_HCO_3_; followed by washing for 30 min with 5% acetic acid in methanol. Following aspiration of wash buffer, the gel pieces were washed additionally in 100 mM NH_4_HCO_3_, followed by aspiration and final wash with acetonitrile. The gel pieces were then dried for 45 min using a SpeedVac vacuum concentrator and subjected to overnight tryptic digestion. Digested peptides were extracted from the gel pieces using 20 μL of a 1:1 mix of 0.1% TFA in H_2_0/acetonitrile for 15 min; followed by spin and supernatant harvest and a second extraction using 20 μL of a 1:2 mix 0.1% TFA in H_2_0/acetonitrile for 15 min. The pooled supernatants were vacuum concentrated to dryness and stored at -80°C for subsequent LC–MS/MS analysis.

EV-derived peptides were separated by reversed-phase chromatography using an EASY*nano* HPLC system coupled online with a LTQ-Orbitrap XL mass spectrometer (Thermo Scientific). Mass spectrometer parameters were: spray voltage 2.5 kV; capillary temperature 280°C; FT resolution 60,000; FT target value 1×10^6^; LTQ target value 3×10^4^; 1 FT microscan with 500 ms injection time; and 1 LTQ microscan with 10 ms injection time. Mass spectra were acquired in a data-dependent mode with the *m*/*z* range of 400-2000. The full mass spectrum (MS scan) was acquired by the FT and tandem mass spectrum (MS/MS scan) was acquired by the LTQ with a 35% normalized collision energy. Acquisition of each full mass spectrum was followed by the acquisition of MS/MS spectra for the five most intense +2 or +3 ions within a one-second duty cycle. The minimum signal threshold (counts) for a precursor occurring during a MS scan was set at 1000 for triggering a MS/MS scan.

The acquired LC-MS/MS data was processed by the Proteome Discoverer 1.4 (Thermo Scientific). The Sequest HT was used as a search engine with the parameters including cysteine (Cys) alkylated with acrylamide (71.03714@C) as a fixed modification and methionine (Met) oxidation (15.99491@M) as a variable modification. Data was searched against the Uniprot human database (November 2015) and further filtered at False Discovery Rate (FDR) of ≤5%.

### Mass spectrometry analysis of human plasma samples

Quantitative mass spectrometry analysis of unfractionated (non-EV-enriched) human plasma samples was performed using a liquid-phase-based orthogonal multidimensional intact-protein analysis system (IPAS) approach as previously described [Wang H, Hanash S. Intact-Protein Analysis System for Discovery of Serum-Based Disease Biomarkers. In: Simpson R, Greening D (eds) Serum/Plasma Proteomics. Methods in Molecular Biology (Methods and Protocols), vol 728. Humana Press (2011)]. Briefly, pooled plasma samples (300 μL) from control and case were processed using immunodepletion chromatography to remove the high-abundance proteins; the remaining protein mass was concentrated and isotopically labeled with ^12^C3- or ^13^C3-acrylamide. Labeled intact-protein samples were mixed and orthogonally separated by 2D-HPLC via anion-exchange (AEX) followed by reversed-phase (RP) chromatography. Collected protein fractions from the 2D-HPLC were then lyophilized and subjected to in-solution tryptic digestion for subsequent LC–MS/MS analysis.

Plasma peptides were separated by reversed-phase chromatography using a *nano* HPLC system (Eksigent) coupled online with a LTQ-Orbitrap XL mass spectrometer (Thermo Scientific). Mass spectrometer parameters were spray voltage 2.5 kV; capillary temperature 200°C; FT resolution 60,000; FT target value 8×10^5^; LTQ target value 10^4^; 1 FT microscan with 850 ms injection time; and 1 LTQ microscan with 100 ms injection time. Mass spectra were acquired in a data-dependent mode with the *m*/*z* range of 400-2000. The full mass spectrum (MS scan) was acquired by the FT and tandem mass spectrum (MS/MS scan) was acquired by the LTQ with a 35% normalized collision energy. Acquisition of each full mass spectrum was followed by the acquisition of MS/MS spectra for the five most intense +2 or +3 ions within a one second duty cycle. The minimum signal threshold (counts) for a precursor occurring during a MS scan was set at 1000 for triggering a MS/MS scan.

The acquired LC-MS/MS data was processed by the Computational Proteomics Analysis System [^*^Rauch et al.]. Briefly, LC-MS/MS data were first converted to mzXML format using ReAdW software (version 1.2) to generate the peak list for protein database searching. The X!Tandem search engine (version 2005.12.01) parameters included cysteine (Cys) alkylated with iodoacetamide (57.02146@C) as a fixed modification and methionine (Met) oxidation (15.99491@M) as a variable modification. Data was searched against the International Protein Index (IPI) human protein knowledgebase (version v3.57). The minimum criterion for peptide matching was a Peptide Prophet Score ≥ 0.2. Peptides meeting this criterion were grouped to protein sequences using the Protein Prophet algorithm at an error rate of ≤5%. Total mass spectrometry counts for each protein were used as a measure of protein abundance. For dataset integration, protein identifiers were collapsed by gene name. [^*^Rauch A, Bellew M, Eng J, et al. Computational Proteomics Analysis System (CPAS): An extensible, open-source analytic system for evaluating and publishing proteomic data and high throughput biological experiments. *Journal of proteome research.* Jan 2006;5(1):112-121.]

### Statistical and bioinformatics analyses

Raw assay data were log2-transformed. A one-sided Wilcoxon rank-sum test was used to compute *P* values comparing lung adenocarcinoma cases with healthy controls. Unsupervised hierarchical clustering was performed by Ward's method to form groups such that the aggregrate within-group sum of squares error was minimized; Pearson correlation was then used as the basis for distance measure. Analyses and plots were implemented in the R software environment (version 3.2.3) and Bioconductor packages (version 3.2). Gene ontology (GO) enrichment analysis was performed using the multiple protein search interface of the STRING database (version 10.0) to evaluate known and predicted protein relationships. Ingenuity pathway analysis (IPA) was used to further explore feature relationships, independent of established canonical pathways according to the associations contained within the Ingenuity Knowledge Base.

## SUPPLEMENTARY MATERIALS FIGURES AND TABLES




