# Supplementary material for: Plasma-derived extracellular vesicle proteins as a source of biomarkers for lung adenocarcinoma
Source: Oncotarget. 2017 Sep 8;8(56):95466–80. doi: 10.18632/oncotarget.20748 (PMC5707035; doi:10.18632/oncotarget.20748)
Supplement: Supplementary file 1 [file oncotarget-08-95466-s001.pdf]

Plasma-derived extracellular vesicle proteins as a source of biomarkers for lung adenocarcinoma

SUPPLEMENTARY MATERIALS

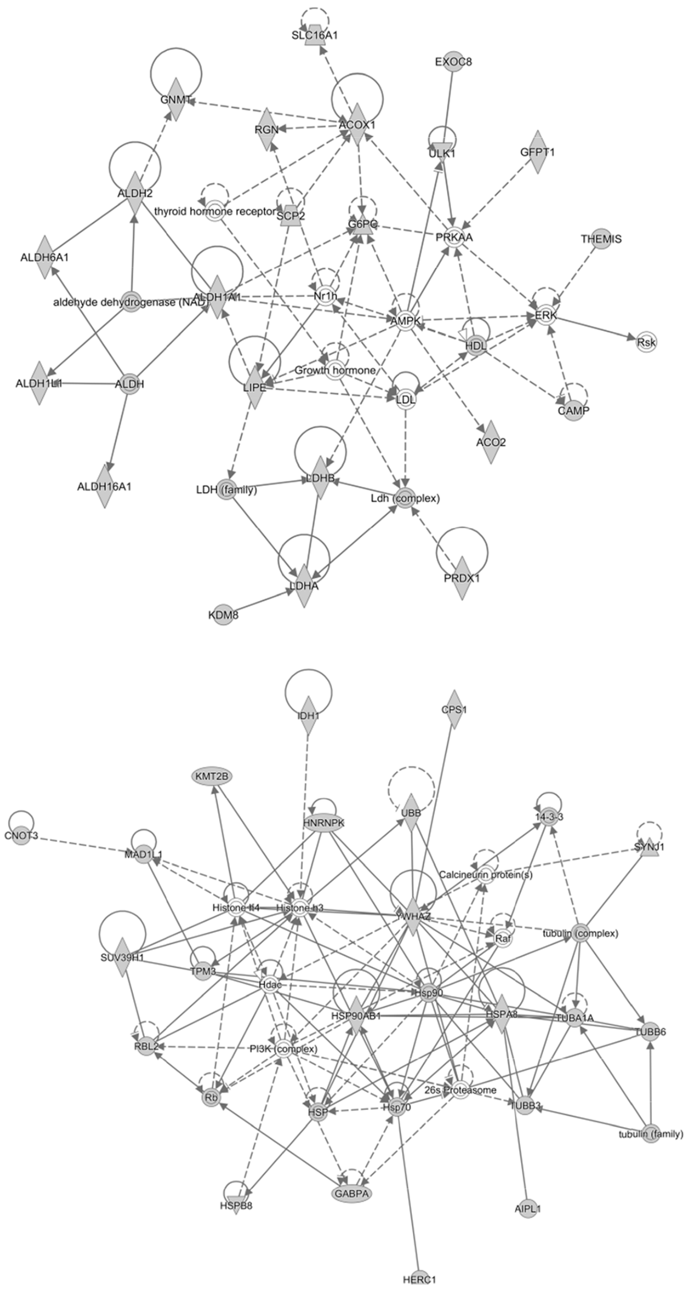

Supplementary Figure 1: IPA network analysis of 203 proteins with > 1.12-fold higher average expression in adenocarcinoma patient case compared to control plasma-derived EVs. The top two scoring IPA networks (*P-score*=10^{-39} and *P-score*=10^{-37}) integrate nodes associated with lipid metabolism, small molecule biochemistry, and cellular movement; as well as cancer, organismal injury and abnormalities, and respiratory disease.

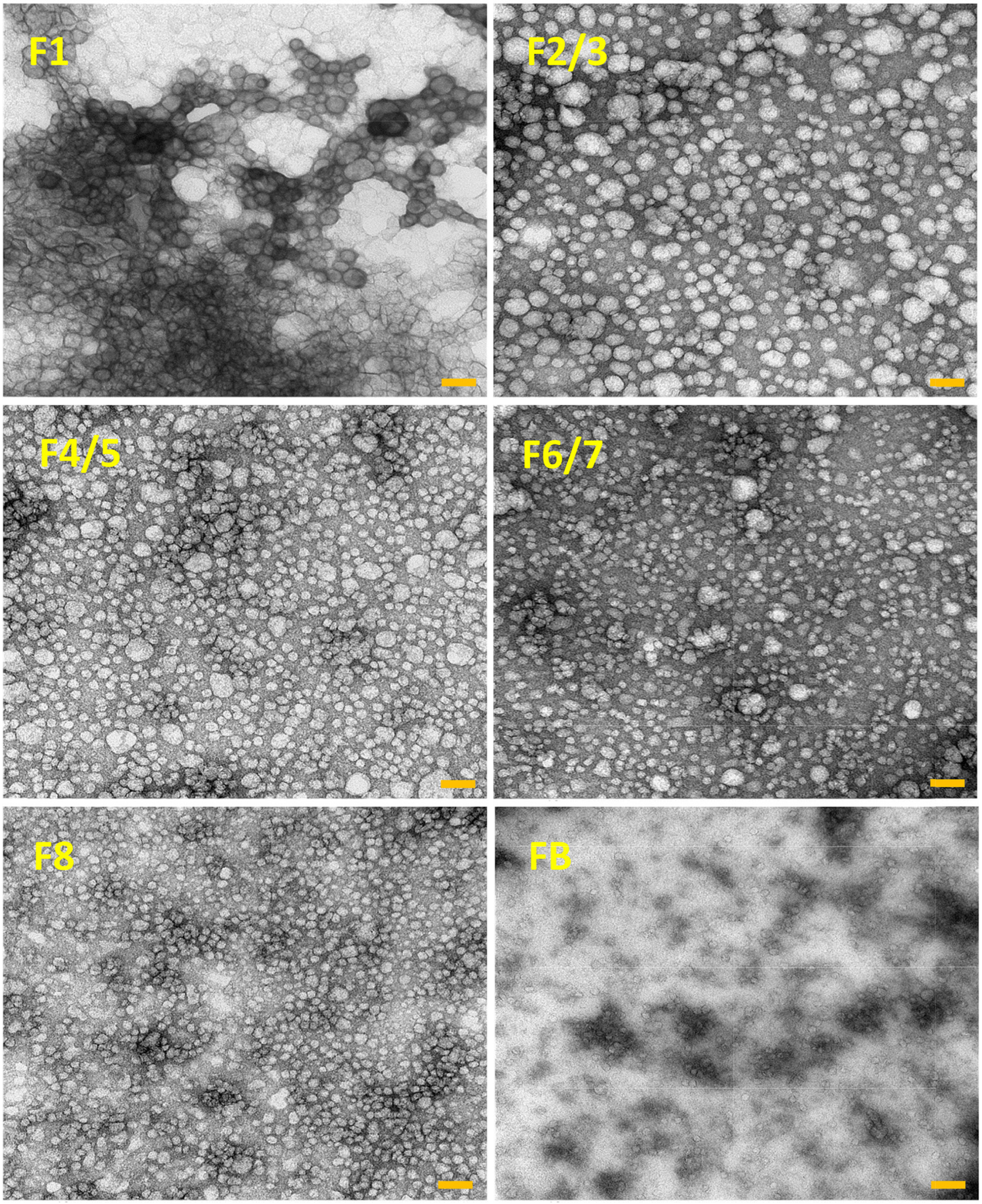

Supplementary Figure 2: Transmission electron microscopy images of plasma-derived EVs obtained from multi-step density fractionation of plasma (scale bar, 100 nm). Fraction numbers correspond with those specified in Figure 1.

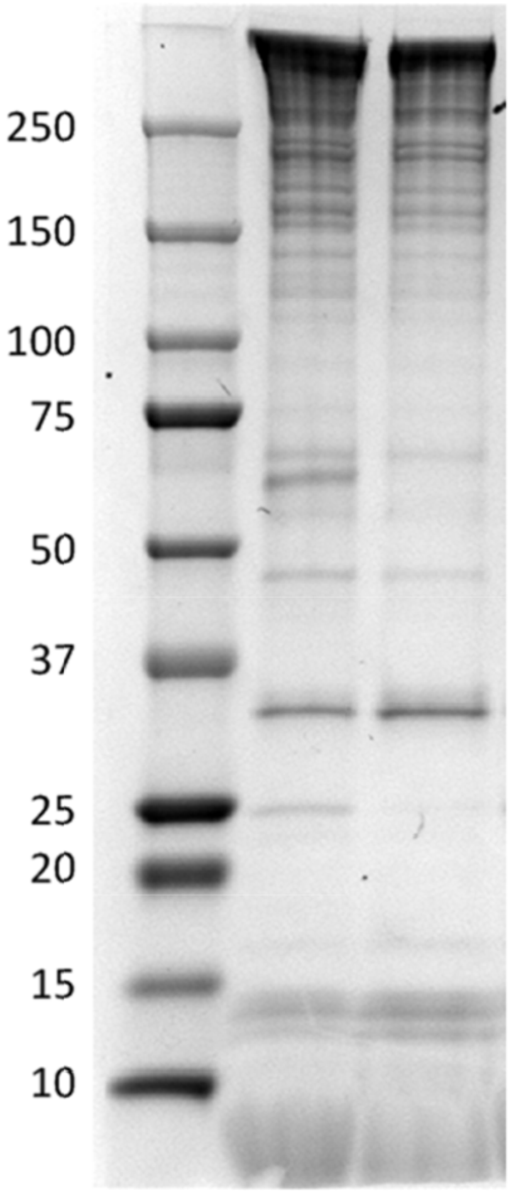

Supplementary Figure 3: Total protein content of representative plasma-derived EVs isolated by ultracentrifugation flotation through a single-step density-overlay (ρ = 1. 14 g/mL) over iodixanol-densified plasma. Lane 1 is molecular weight marker; Lane 2 is vesicle harvest following density flotation; Lane 3 is vesicle harvest resulting from two cycles of density flotation (Lane 2 harvest was used as input for serial re-isolation run).

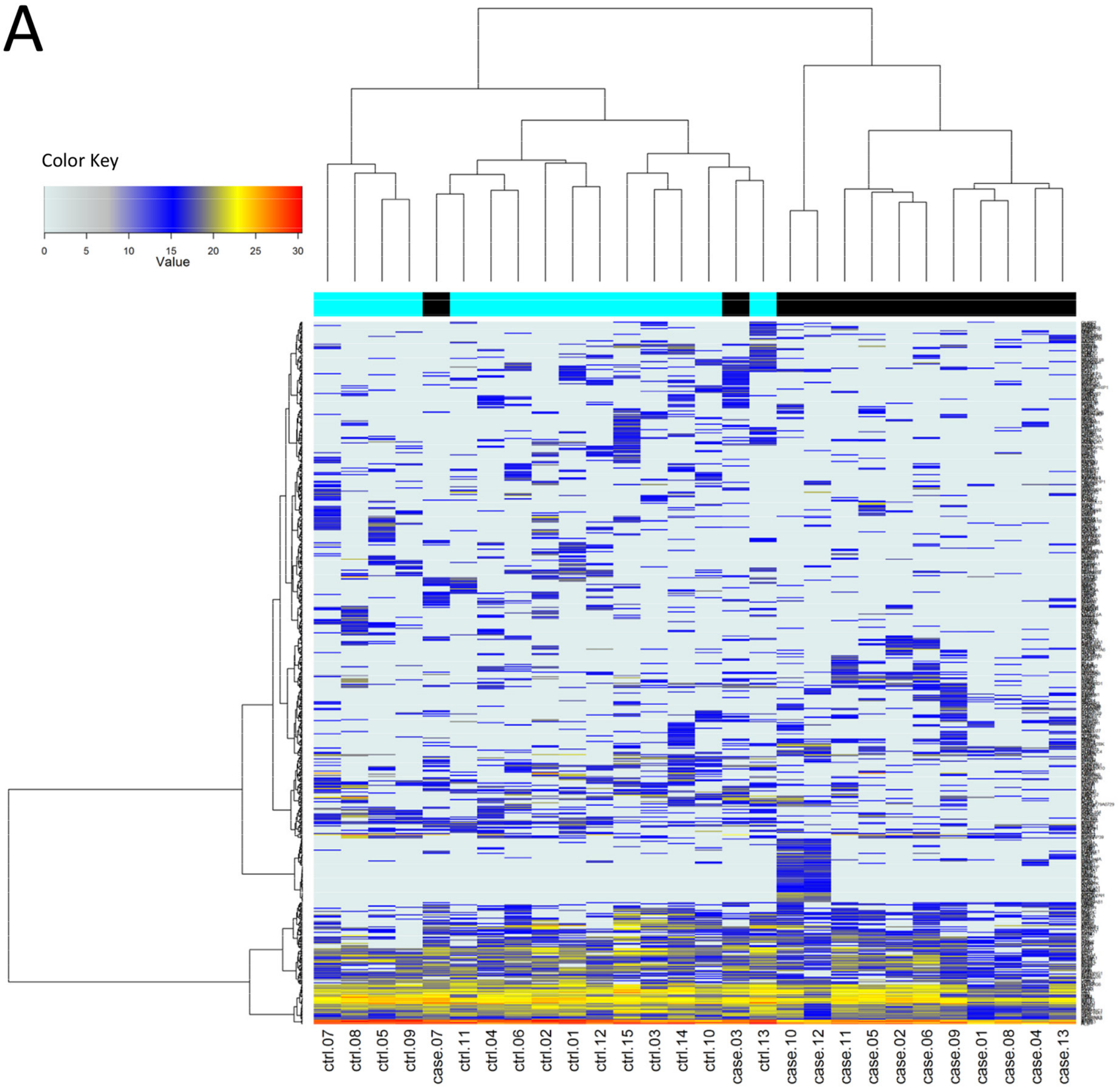

Supplementary Figure 4: Hierarchical clustering analyses of plasma-derived EV proteins. Lung adenocarcinoma cases are in black; disease free controls are in cyan. From Figure 2A: Intensity heat map of all proteins identified by LC MS/MS proteomics.

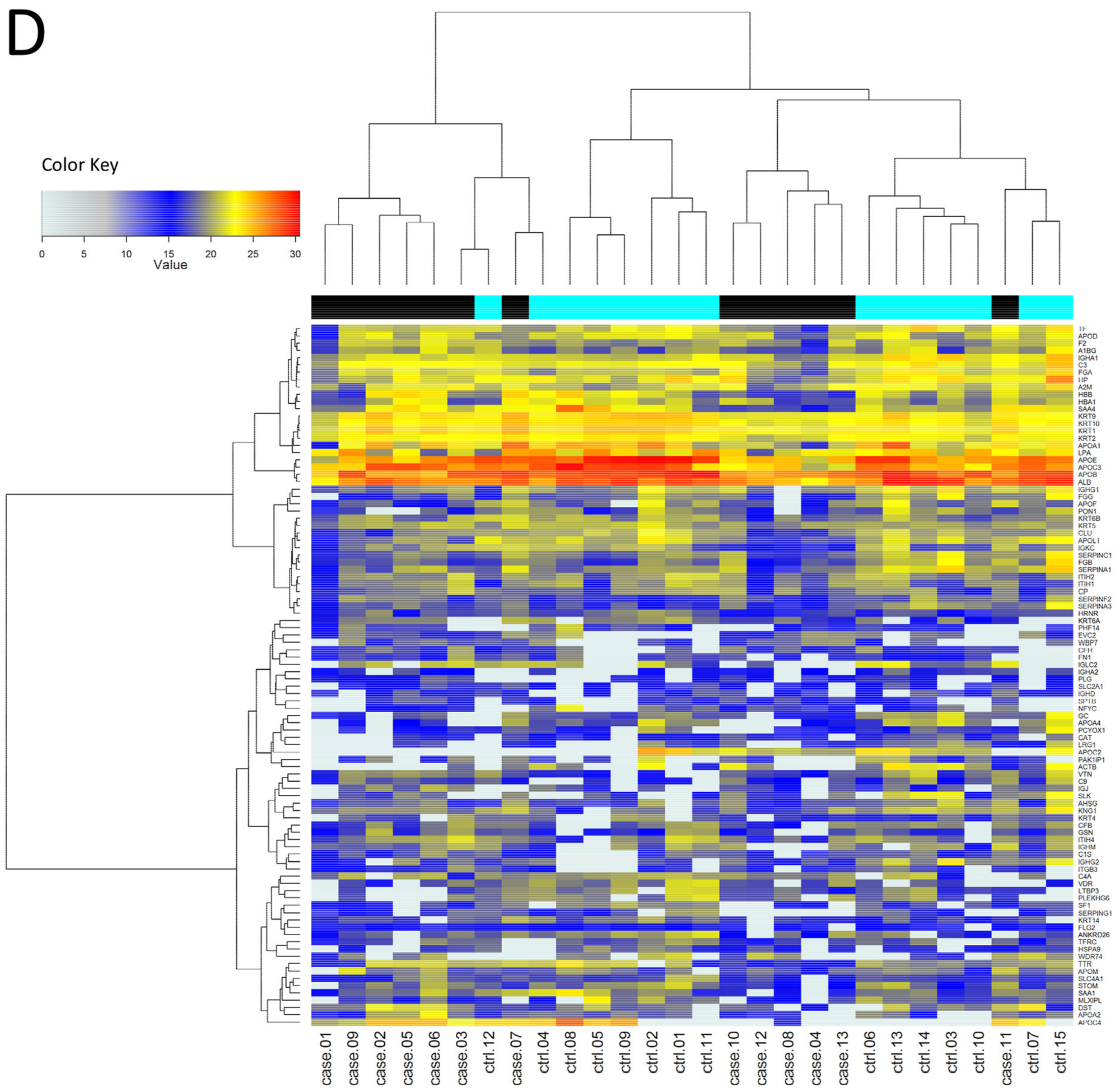

Supplementary Figure 5: Hierarchical clustering analyses of plasma-derived EV proteins. Lung adenocarcinoma cases are in black; disease free controls are in cyan. From Figure 2D: Intensity heat map of high abundance protein group.

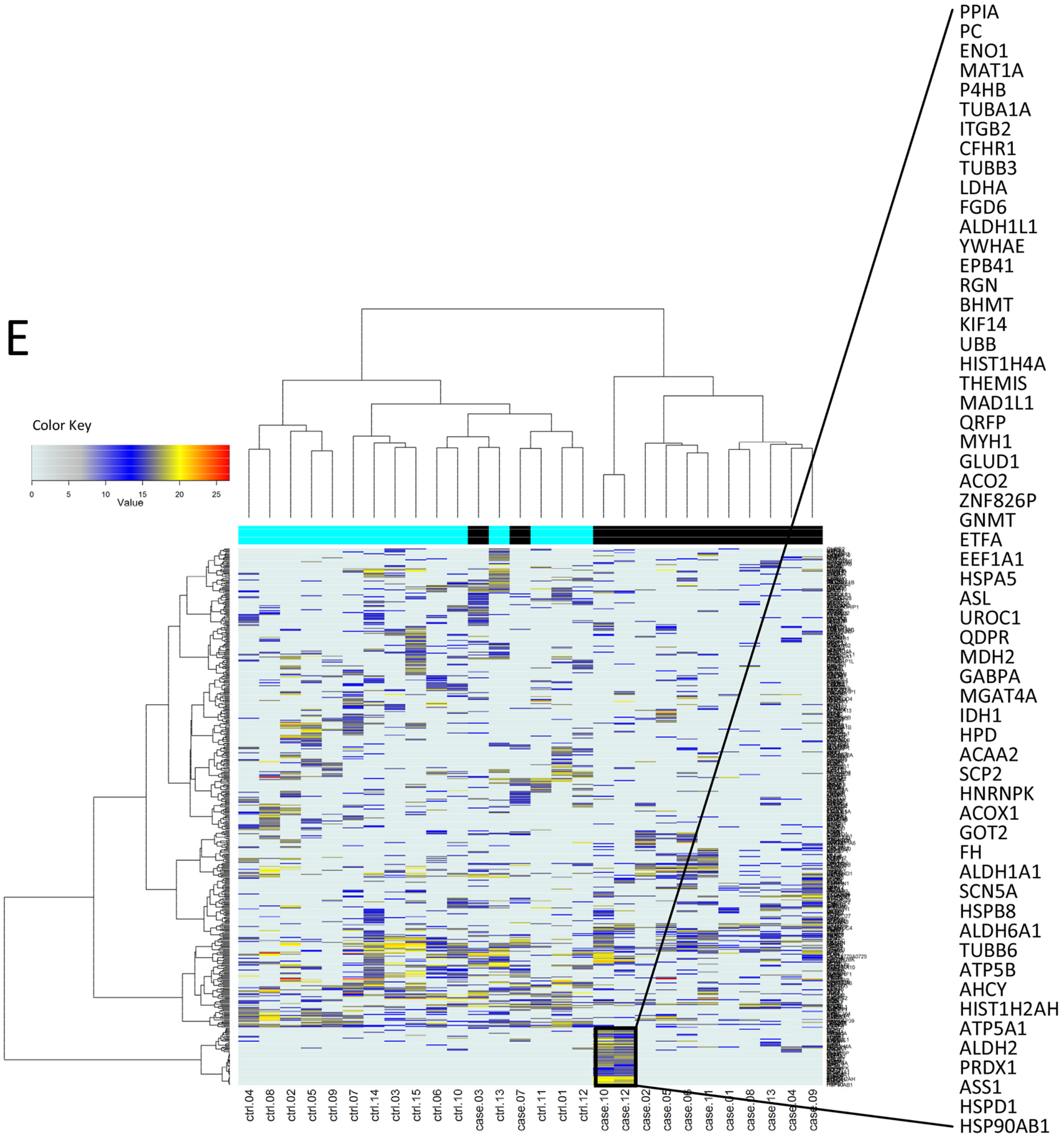

Supplementary Figure 6: Hierarchical clustering analyses of plasma-derived EV proteins. Lung adenocarcinoma cases are in black; disease free controls are in cyan. From Figure 2E: Intensity heat map of low abundance protein group; a cancer-related cluster is highlighted.

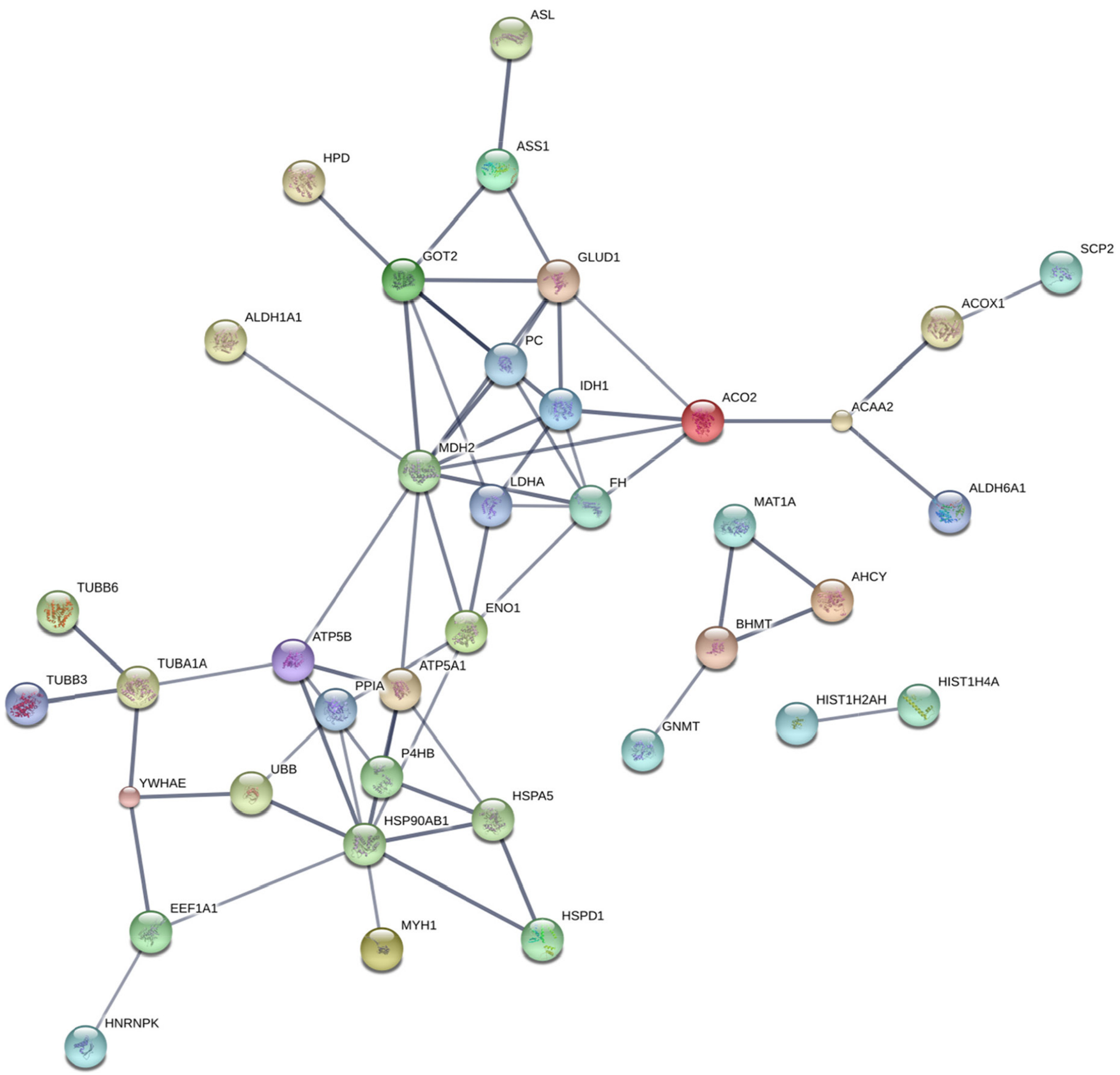

Supplementary Figure 7: STRING Protein-protein interaction network connectivity for lung adenocarcinoma plasma-derived EV protein cluster highlighted in Supplementary Figure 6. Network contains 62 edges (vs. 11 expected edges); enrichment p-value< 0.001. Confidence score threshold was set at 0.7 (high).

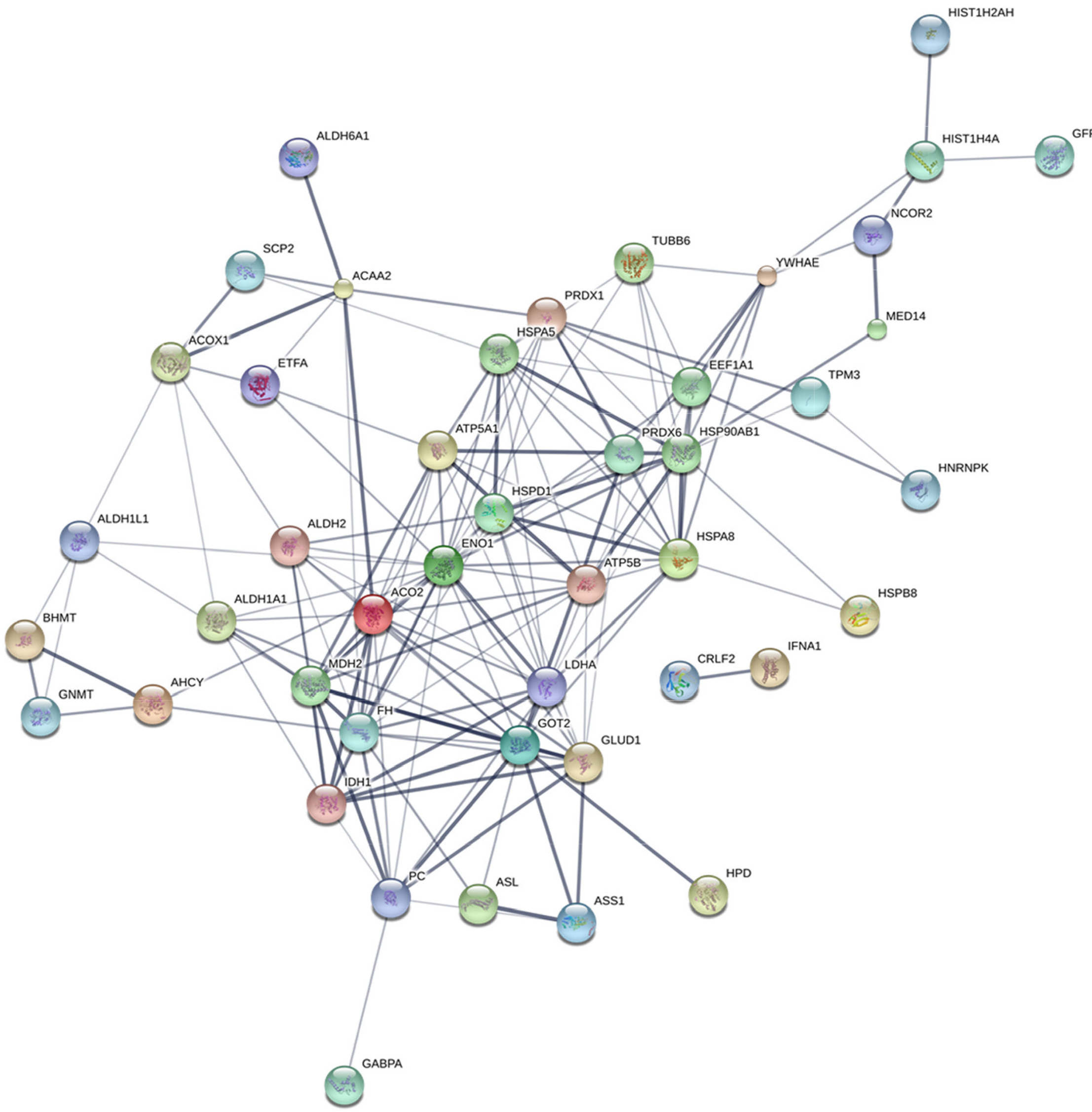

Supplementary Figure 8: STRING Protein-protein interaction network connectivity for adenocarcinoma case EV-associated proteins exhibiting > 2-fold higher mean expression compared to controls. Network contains 75 nodes with 160 edges (vs. 38 expected edges); clustering coefficient: 0.742; enrichment p-value < 0.001. Confidence score threshold was set at 0.7 (high).

Supplementary Table 1: Differentially expressed (case:control >2-fold) adenocarcinoma plasma EV-associated proteins with corresponding expression in EVs derived from lung cancer cell lines H23, H647, H1573, HCC4019

See Supplementary File 1

Supplementary Table 2A: Enrichment analysis. GO cellular component terms. Adenocarcinoma case EVs, (case:control > 2-fold)

Pathway ID	Pathway description	Gene count	FDR	Matching proteins in network
GO.0070062	*extracellular exosome*	38	1.41E-12	ACAA2, AHCY, ALDH1A1, ALDH1L1, ALDH2, ALDH6A1, ASL, ASS1, ATP5A1, ATP5B, BHMT, ENO1, ETFA, FH, GFPT1, GOT2, HIST1H2AH, HIST1H4A, HNRNPK, HPD, HSP90AB1, HSPA5, HSPA8, HSPD1, HUWE1, IDH1, ITGB2, KIF27, LDHA, MGAT4A, NCCRP1, PRDX1, PRDX6, QDPR, SCP2, TPM3, TUBB6, YWHAE
GO.0005576	*extracellular region*	42	1.47E-09	ACAA2, AHCY, ALDH1A1, ALDH1L1, ALDH2, ALDH6A1, ASL, ASS1, ATP5A1, ATP5B, BHMT, CRLF2, CTRL, ENO1, ETFA, FH, GFPT1, GOT2, HIST1H2AH, HIST1H4A, HNRNPK, HPD, HSP90AB1, HSPA5, HSPA8, HSPD1, HUWE1, IDH1, IFNA1, ITGB2, KIF27, LDHA, MGAT4A, NCCRP1, PRDX1, PRDX6, QDPR, SCP2, SRGN, TPM3, TUBB6, YWHAE
GO.0005739	*mitochondrion*	22	2.40E-06	ACO2, ACOX1, ALDH1L1, ALDH2, ALDH6A1, ASS1, ATP5A1, ATP5B, ETFA, FH, GLUD1, HNRNPK, HSP90AB1, HSPA5, HSPD1, IDH1, LDHA, PC, PRDX1, QDPR, SCP2, YWHAE
GO.0005759	*mitochondrial matrix*	11	8.62E-06	ACO2, ALDH2, ALDH6A1, ATP5A1, ATP5B, ETFA, FH, GLUD1, GOT2, HSPD1, PC

Supplementary Table 2B: Enrichment analysis. GO molecular function terms. Adenocarcinoma case EVs, (case:control > 2-fold)

Pathway ID	Pathway description	Gene count	FDR	Matching proteins in network
GO.0048037	*cofactor binding*	9	6.72E-04	AHCY, ALDH6A1, ETFA, GLUD1, GOT2, IDH1, LDHA, QDPR, SCP2
GO.0050662	*coenzyme binding*	8	6.72E-04	AHCY, ALDH6A1, ETFA, GLUD1, IDH1, LDHA, QDPR, SCP2
GO.0003824	*catalytic activity*	37	9.80E-04	ACAA2, ACO2, AHCY, ALDH1A1, ALDH1L1, ALDH2, ASL, ASS1, BHMT, CTRL, EEF1A1, ENO1, ETFA, FH, GFPT1, GLUD1, GNMT, GOT2, HIST1H4A, HPD, HSPA5, HSPA8, HSPD1, HUWE1, IDH1, KIF14, KIF27, LDHA, MGAT4A, PLCE1, PRDX1, PRDX6, QDPR, RGN, SCP2, TUBB6, UROC1
GO.0016491	*oxidoreductase activity*	12	1.72E-03	ALDH1A1, ALDH1L1, ALDH2, ALDH6A1, ETFA, GLUD1, HPD, IDH1, LDHA, PRDX1, PRDX6, QDPR
GO.0031406	*carboxylic acid binding*	7	1.72E-03	ASS1, GLUD1, GNMT, GOT2, PC, SCP2, YWHAE
GO.0023026	*MHC class II protein complex binding*	3	2.99E-03	HSP90AB1, HSPA8, YWHAE
GO.0044822	*poly(A) RNA binding*	15	3.74E-03	ACAA2, ALDH6A1, ASS1, ATP5A1, CAPRIN1, ENO1, GOT2, HIST1H4A, HNRNPK, HSP90AB1, HSPA8, HSPD1, HUWE1, PRDX1, YWHAE
GO.0016597	*amino acid binding*	5	4.90E-03	ASS1, GLUD1, GNMT, GOT2, YWHAE
GO.0036094	*small molecule binding*	23	4.90E-03	AHCY, ASS1, ATP5A1, ATP5B, EEF1A1, ETFA, GLUD1, GNMT, GOT2, HSP90AB1, HSPA5, HSPA8, HSPD1, IDH1, KIF14, KIF27, LDHA, NIN, PC, QDPR, SCP2, TUBB6, YWHAE
GO.0016836	*hydro-lyase activity*	4	5.20E-03	ACO2, ENO1, FH, UROC1
GO.0019899	*enzyme binding*	15	1.02E-02	EEF1A1, ENO1, EXOC8, HNRNPK, HSP90AB1, HSPA5, HSPA8, HSPD1, ITGB2, KIF14, NCOR2, PLCE1, PRDX6, SCN5A, YWHAE
GO.0031625	*ubiquitin protein ligase binding*	6	2.37E-02	HSPA5, HSPA8, HSPD1, PRDX6, SCN5A, YWHAE

Supplementary Table 3: Stage information for cases used to test TPM3 performance by immunoblotting of EVs derived from cases and controls and reported in Figure 4C

Lane	Lung Carcinoma Subtype	T	N	M	Stage
1	Adenocarcinoma	T2a	N0	M0	IB
2	Adenocarcinoma	T1b	N0	M0	IA
3	Squamous Cell Carcinoma	T2a	N1	M0	IIA
4	Adenocarcinoma	T2a	N0	M0	IB
5	Adenocarcinoma	T2a	N1	M0	IIA
6	Adenocarcinoma	T2a	N1	M0	IIA
7	Adenocarcinoma	T1b	N0	M0	IB
8	Adenocarcinoma	T3	N0	M0	IIB
9	Adenocarcinoma	T2a	N0	M0	IB
10	Adenocarcinoma	T3	N0	M0	IIB
11	Adenocarcinoma	T1a	N1	M0	IIA
12	Adenosquamous carcinoma	T1b	N1	M0	IIA
