# Supplementary material for: Plasma-derived extracellular vesicle proteins as a source of biomarkers for lung adenocarcinoma
Source: Oncotarget. 2017 Sep 8;8(56):95466–80. doi: 10.18632/oncotarget.20748 (PMC5707035; doi:10.18632/oncotarget.20748)
Supplement: Supplementary file 2 [file oncotarget-08-95466-s002.docx]

**Supplementary Table 1:** **Differentially expressed (case:control >2-fold) adenocarcinoma plasma EV-associated proteins with corresponding expression in EVs derived from lung cancer cell lines H23, H647, H1573, HCC4019**

|  | **Mean relative ion intensity** | |  |  |
| --- | --- | --- | --- | --- |
| **Gene** | **Control EVs** | **Case EVs** | **Fold change (case:ctrl)** | **Cell line** |
| NCCRP1 | 0.00E+00 | 1.06E+04 | *inf* | x |
| ALDH1L1 | 0.00E+00 | 1.14E+05 | *inf* | x |
| HIST1H4A | 0.00E+00 | 6.60E+04 | *inf* | x |
| MED14 | 0.00E+00 | 7.00E+03 | *inf* | x |
| PPIA | 0.00E+00 | 1.89E+04 | *inf* | x |
| GLUD1 | 0.00E+00 | 5.24E+04 | *inf* | x |
| BHMT | 0.00E+00 | 7.71E+04 | *inf* | x |
| EXOC8 | 0.00E+00 | 5.58E+03 | *inf* | x |
| ATP5B | 0.00E+00 | 1.83E+05 | *inf* | x |
| AHCY | 0.00E+00 | 1.57E+05 | *inf* | x |
| ATP5A1 | 0.00E+00 | 1.25E+05 | *inf* | x |
| ALDH2 | 0.00E+00 | 9.56E+04 | *inf* | x |
| HSPD1 | 0.00E+00 | 7.38E+04 | *inf* | x |
| HSP90AB1 | 0.00E+00 | 7.05E+04 | *inf* | x |
| PRDX1 | 0.00E+00 | 6.76E+04 | *inf* | x |
| XPO4 | 0.00E+00 | 5.05E+04 | *inf* | x |
| KIF27 | 0.00E+00 | 3.51E+04 | *inf* | x |
| HSPA5 | 0.00E+00 | 3.49E+04 | *inf* | x |
| YWHAE | 0.00E+00 | 2.16E+04 | *inf* | x |
| ASL | 0.00E+00 | 2.05E+04 | *inf* | x |
| ETFA | 0.00E+00 | 1.98E+04 | *inf* | x |
| CNOT3 | 0.00E+00 | 1.98E+04 | *inf* | x |
| EEF1A1 | 0.00E+00 | 1.83E+04 | *inf* | x |
| IDH1 | 0.00E+00 | 1.64E+04 | *inf* | x |
| MDH2 | 0.00E+00 | 1.38E+04 | *inf* | x |
| QDPR | 0.00E+00 | 1.20E+04 | *inf* | x |
| ACAA2 | 0.00E+00 | 1.18E+04 | *inf* | x |
| ALDH1A1 | 0.00E+00 | 1.07E+04 | *inf* | x |
| NCOR2 | 0.00E+00 | 8.47E+03 | *inf* | x |
| GFPT1 | 0.00E+00 | 8.41E+03 | *inf* | x |
| FH | 0.00E+00 | 7.70E+03 | *inf* | x |
| HSPB8 | 0.00E+00 | 6.74E+03 | *inf* | x |
| TUBB6 | 0.00E+00 | 5.60E+03 | *inf* | x |
| HNRNPK | 0.00E+00 | 4.27E+03 | *inf* | x |
| UBB | 4.40E+02 | 6.13E+04 | 139.31 | x |
| CPS1 | 1.20E+04 | 8.55E+05 | 71.47 | x |
| ENO1 | 4.03E+02 | 2.43E+04 | 60.29 | x |
| KRT27 | 5.12E+02 | 2.50E+04 | 48.80 | x |
| TPM3 | 6.05E+02 | 2.67E+04 | 44.10 | x |
| EPB41 | 1.42E+03 | 3.29E+04 | 23.18 | x |
| P4HB | 3.30E+03 | 5.10E+04 | 15.45 | x |
| ULK1 | 1.43E+04 | 2.12E+05 | 14.90 | x |
| RAP1B | 1.76E+03 | 2.38E+04 | 13.47 | x |
| PRDX6 | 8.14E+02 | 1.07E+04 | 13.19 | x |
| PSMA6 | 2.54E+02 | 3.17E+03 | 12.48 | x |
| HSPA8 | 8.98E+03 | 1.02E+05 | 11.42 | x |
| CCDC18 | 6.38E+03 | 6.24E+04 | 9.77 | x |
| FBLN1 | 5.15E+02 | 4.74E+03 | 9.22 | x |
| HUWE1 | 1.12E+04 | 9.36E+04 | 8.36 | x |
| XPO6 | 8.72E+02 | 6.17E+03 | 7.08 | x |
| NKX2-1 | 2.98E+03 | 2.04E+04 | 6.84 | x |
| MAD1L1 | 1.29E+04 | 8.49E+04 | 6.60 | x |
| HBD | 7.05E+04 | 4.43E+05 | 6.28 | x |
| MAT1A | 2.07E+03 | 1.23E+04 | 5.96 | x |
| AKT2 | 8.25E+03 | 4.58E+04 | 5.55 | x |
| SRGN | 8.69E+03 | 4.61E+04 | 5.30 | x |
| THBS1 | 5.37E+03 | 2.83E+04 | 5.27 | x |
| MYH9 | 1.38E+04 | 6.77E+04 | 4.92 | x |
| MADD | 3.97E+03 | 1.67E+04 | 4.21 | x |
| KRT16 | 1.75E+04 | 7.29E+04 | 4.15 | x |
| LDHA | 2.94E+03 | 1.21E+04 | 4.12 | x |
| TUBA1A | 5.64E+03 | 2.27E+04 | 4.02 | x |
| PIGR | 5.41E+03 | 2.14E+04 | 3.95 | x |
| CYBRD1 | 7.36E+03 | 2.81E+04 | 3.82 | x |
| ACTA1 | 1.80E+04 | 6.55E+04 | 3.64 | x |
| C1R | 2.79E+03 | 1.01E+04 | 3.60 | x |
| SLC16A1 | 9.89E+02 | 3.17E+03 | 3.20 | x |
| PLG | 2.70E+04 | 7.35E+04 | 2.72 | x |
| APEH | 5.62E+03 | 1.39E+04 | 2.47 | x |
| HLA-B | 4.16E+03 | 9.62E+03 | 2.31 | x |
| PDLIM1 | 8.10E+03 | 1.82E+04 | 2.25 | x |
| RANBP10 | 9.18E+03 | 2.03E+04 | 2.21 | x |
| TUBB3 | 2.56E+03 | 5.60E+03 | 2.19 | x |
| LACTB2 | 7.80E+03 | 1.56E+04 | 2.00 | x |
